# Assembling Bare Au Nanoparticles at Positively Charged Templates

**DOI:** 10.1038/srep26462

**Published:** 2016-05-26

**Authors:** Wenjie Wang, Honghu Zhang, Ivan Kuzmenko, Surya Mallapragada, David Vaknin

**Affiliations:** 1 Division of Materials Sciences and Engineering, Ames Laboratory, USDOE, Ames, Iowa 50011, United States; 2 Department of Materials Science and Engineering, Iowa State University, Ames, Iowa 50011, United States; 3 X-ray Science Division, Advanced Photon Source, Argonne National Laboratory, Lemont, Illinois 60439, United States; 4 Department of Chemical and Biological Engineering, Iowa State University, Ames, Iowa 50011, United States; 5 Department of Physics and Astronomy, Iowa State University, Ames, Iowa 50011, United States

## Abstract

*In-situ* X-ray reflectivity (XRR) and grazing incidence X-ray small-angle scattering (GISAXS) reveal that unfunctionalized (bare) gold nanoparticles (AuNP) spontaneously adsorb to a cationic lipid template formed by a Langmuir monolayer of DPTAP (1,2-dihexadecanoyl-3-trimethylammonium-propane) at vapor/aqueous interfaces. Analysis of the XRR yields the electron density profile across the charged-interfaces along the surface normal showing the AuNPs assemble with vertical thickness comparable to the particle size. The GISAXS analysis indicates that the adsorbed mono-particle layer exhibits short-range in-plane correlations. By contrast, single-stranded DNA-functionalized AuNPs, while attracted to the positively charged surface (more efficiently with the addition of salt to the solution), display less in-plane regular packing compared to bare AuNPs.

Langmuir monolayers assembled with insoluble, amphiphilic organic molecules, exhibiting rich phase behaviors, have been studied extensively as a model two dimensional (2D) systems for decades^1^. Surface X-ray scattering techniques, such as specular reflectivity (XRR) and grazing incidence diffraction (GIXD), have played a decisive role in relating the phase behaviors, generally obtained from surface-pressure versus molecular area (*π* − *A*) isotherms, to the structure of the monolayer at unprecedented molecular length scales^2^. The 2D nature of orderly Langmuir monolayers has inspired their use as templates to induce the growth of 2D organic and inorganic crystals^3^. On different length scales, 2D reversible clustering, and ordering of uniform colloidal particles have been accomplished by trapping them at air/water interfaces^4^,^5^,^6^,^7^. Subsequently, charged vesicle surfaces have been used to assemble colloidal particles in solutions^8^. More recently, NP self-assembly has been achieved and even though at times straightforward, it can be complicated by the interplay between these particles and their immediate environment that can be different than that in the bulk-medium (i.e., pH and salt concentrations near NP surfaces)^9^,^10^,^11^. In fact, the same properties are used to disperse and stabilize AuNPs in aqueous solutions, either by electrostatic repulsion or steric forces^11^. Spontaneous 2D assembly and crystallization of functionalized (capped) AuNPs with ssDNA by adjusting salt concentrations have been reported recently^12^,^13^,^14^. Similarly ssDNA-capped-AuNP have been assembled and crystallized at charged Langmuir monolayers with the goal to produce 2D meta-materials as an alternative to common top-down lithographical approach^15^,^16^. In this study, we explore the interplay of the two key components of template-directed self-assembly, AuNPs as building blocks and a cationic lipid monolayer, i.e. 1,2-dihexadecanoyl-3-trimethylammonium-propane (DPTAP), as the template. The motivation of this two-dimensional model-system study is to explore underlying control parameters that can be exploited by design to assemble regular structures from nano-particles. We also demonstrate the application of synchrotron based X-ray liquid surface scattering techniques, i.e., specular reflectivity and grazing incidence scattering, and provide analytical tools to analyze similar GISAXS data in general.

## Results and Discussion

### Small-angle X-ray scattering (SAXS)

Solution SAXS provides the actual size information of independent particles suspended in dilute solutions. The single AuNP is modeled as a solid sphere of radius *R*. Its scattering intensity can be expressed as 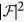, where 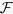 is the form factor for a solid sphere of excess scattering length density, Δ*ρ*, expressed as^17^


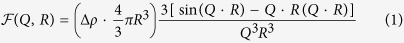


The 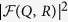 function features periodic variations in *Q*, where maxima occur at 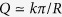, with *k* being a positive integer^18^.

**Figure 1:**
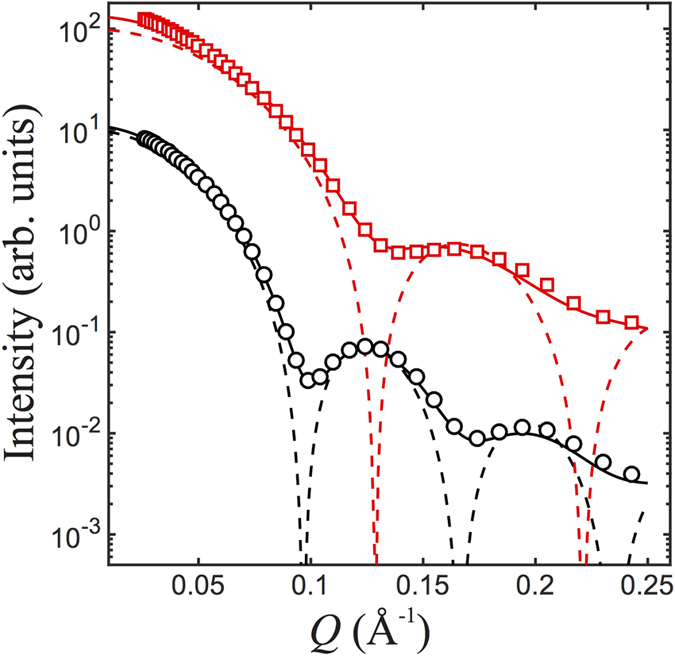
1D SAXS data for suspended AuNPs in solutions. Black circles and red squares represent the data from AuNP of nominal diameter 10 and 5 nm, respectively. The solid lines and dashed lines are calculated intensity profiles from polydisperse and monodisperse spheres, respectively. The intensity data are re-scaled for display purpose.

Figure 1 shows two sets of 1D SAXS data, one (black circles) from AuNP of nominal diameter 10 nm, and the other (red squares) of nominal diameter 5 nm. The nearly periodic variations in intensity are visible in both cases, and are inversely proportional to the particle size, consistent with Eq. (1). The dashed-lines in Fig. 1 are calculated intensity profiles for mono-disperse spheres that capture the intensity maxima to the largest extent. The smeared intensity minima of the SAXS data indicate a modest poly-dispersity in size distribution of AuNPs. Due to the lack of a deterministic way of knowing the polydispersity associated with the AuNPs, we only assume the radii of the AuNPs obey a Gaussian distribution *D*(*R*), i.e., 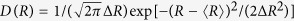, where 〈*R*〉 and Δ*R* represent the mean and the spread of the particle radii, respectively. The total intensity, *I*(*Q*), from such a collection of particles, is expressed as^19^


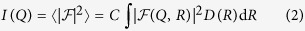


where *C* denotes an intensity scale factor.

Profile-fitting in terms of Eq. (2) provides 〈*R*〉 and Δ*R*, as is summarized in Table 1. The mean diameter for the large particle group is smaller than the nominal value, in contrast to that for the small particle group. According to Eq. (2), the larger the particle, the more weight it carries in the total intensity. The dashed lines in Fig. 1 that match the intensity maxima are calculated for monodisperse spheres of radius approximately equal to 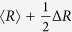. Therefore, the intensity maxima still provide a good estimate of the particle size.

The SAXS intensity profiles of the DNA-coated AuNPs (data not shown), after scaling, are identical to those of bare AuNPs, indicating the X-rays are insensitive to the DNA outer-shell whose electron density is too close to that of the aqueous environment.

### X-ray Reflectivity (XRR)

Insoluble amphiphiles, such as DPTAP, after being spread on aqueous surfaces and compressed into a relatively densely packed state, form a Langmuir monolayer characteristic of stratified structure, i.e., a hydrophobic stratum for its hydrocarbon chains and a hydrophilic stratum for its polarized headgroups^20^. Its vertical, stratified structure, manifested as the electron density (ED) depth profile, *ρ*(*z*), across the interface and along the normal to the aqueous surface, can be determined by the specular XRR technique^17^,^21^. The XRR data, *R*, after being normalized to the calculated Fresnel reflectivity *R*_F_ for an ideally smooth and sharp air-water interface, are related to the corresponding interfacial ED profiles through structural refinement. To this end, a parametrized, continuous ED profile is constructed based on an appropriate structural model and further refined through comparison of its calculated reflectivity to the experimental data^21^,^22^. In this study, the effective-density model is employed for the ED profile construction^22^. The calculation of the reflectivity of a given ED profile is based on the Parratt’s recursive method^17^.

**Figure 2:**
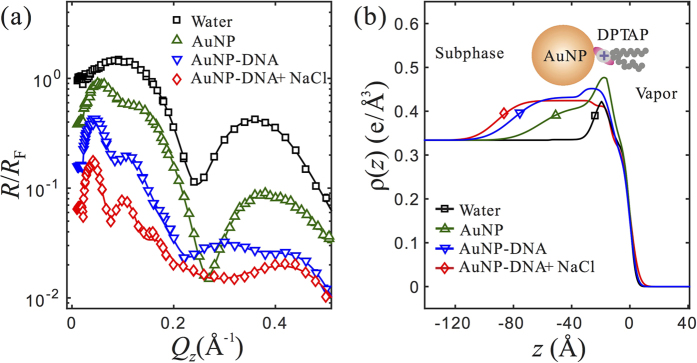
(**a**) *R*/*R*_F_ data for DPTAP monolayers spread on water subphase 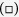, subphase containing AuNPs (10 nm in nominal diameter) of bare surface (Δ), AuNPs (10 nm in nominal diameter) coated with DNA 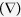 and in the presence of 0.1 M NaCl 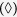. The *R*/*R*_F_ ≈ 1 at *Q*_*z*_ < 0.0218 Å^−1^ and the data are shifted vertically for clarity. Solid lines are best-fit calculated reflectivities based on the refined parameters. (**b**) The corresponding electron density profiles across the air-water interface based on the best-fit structural parameters.

Figure 2 shows the *R*/*R*_F_ data for a DPTAP monolayer on various subphases under otherwise identical conditions of AuNP (10 nm nominal diameter of bare and capped with ssDNA) solutions. The *R*/*R*_F_ data for the DPTAP monolayer on a pure water subphase features fringes with two maxima at *Q*_*z*_ ≈ 0.09 and 0.36 Å^−1^ and the first minimum at *Q*_*z*_ ≈ 0.24 Å^−1^, which is similar to previously obtained XRR for DPTAP on pure water (albeit measured at relative higher surface pressure) using an in-house reflectometer^20^. Qualitatively, if the monolayer is viewed as a single homogeneous slab of thickness *L*, *R*/*R*_F_ fringes are known as the Kiessig fringes corresponding to 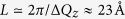, Δ*Q*_*z*_ being the separation of the two consecutive maxima in *Q*_*z*_^17^. In a subtler model, the monolayer is viewed as constituted by two slabs of different ED, i.e. a slab for head groups (of thickness *l*_H_ ≈ 12 Å^20^) and the other for tail groups (of thickness *l*_T_ ≈ 13 Å^20^). In such case, 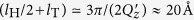, 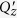 being the first reflectivity minimum in *Q*_*z*_^23^.

The above semi-quantitative estimate is consistent with the corresponding ED profile constructed in terms of the best-fit structural parameters as shown in Fig. 2. On the ED profiles, the subphase, the head group, tail group and the vapor phase are readily recognized. In the presence of the AuNPs (nominal 10 nm diameter) in the subphase, the *R*/*R*_F_ profiles differ significantly from that for a mere water subphase. The first *R*/*R*_F_ maximum shifts to a much lower *Q*_*z*_ approximately 0.04 Å^−1^, indicating the formation of thickened layers. The new *R*/*R*_F_ maxima, lacking obvious periodicity as commonly seen in Kiessig fringes, can only be accounted for by a finer ED depth profiles, as shown in Fig. 2(b). Compared to the ED profile for the DPTAP on water, the presence of AuNPs in the subphase solutions results in significant ED enhancement in an extensive portion across ~50–100 Å immediately below the monolayer-subphase interface. Indeed, at neutral pH DPTAP monolayer is positively charged while surface charge of bare AuNPs is negative with corresponding Zeta potential of −40.6 mV. The bare AuNPs bind to DPTAP monolayer through electrostatic interaction and enrich the surface ED. Considering the diameter of the AuNPs of bare surface is 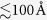, this is evidence that the AuNPs adsorbed underneath the monolayer form a single layer. The DNA surface coating and further the presence of NaCl both facilitate the AuNPs surface adsorption, as evidenced by the relatively more extended in thickness and uniform adsorption layer on the ED profiles. We note that based on the average ED extracted from the X-ray reflectivity we conclude that the surface is not fully covered by AuNPs (assuming the cross section a AuNP is approximately 100 nm^2^, we estimate a 5 to 10% surface coverage consistent with the GISAXS discussed below).

### Grazing incidence small-angle scattering (GISAXS)

**Figure 3:**
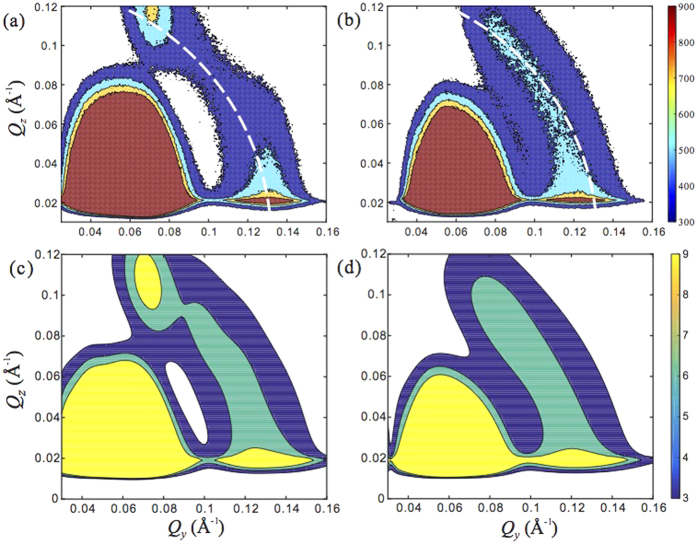
Two-dimensional contour maps for GISAXS intensity as function of *Q*_*z*_ and *Q*_*y*_ for (**a**) AuNPs of bare surface (10 nm in nominal dimater) (**b**) AuNPs (10 nm in nominal diameter) coated with DNA, both with DPTAP monolayers. The white dashed lines are an arc at 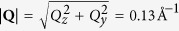. (**c**,**d**) Simulated 2D GISAXS intensity countours for (**a**,**b**), respectively.

The XRR indicates that the surface adsorbed AuNPs is restricted within a single layer. Figure 3 shows two representative GISAXS intensity contour plots as a function of *Q*_*y*_ and *Q*_*z*_. Both contours feature an intensity maximum at low *Q*_*y*_ − *Q*_*z*_ regime, of which the origin is to be explored. There is also a second intensity maximum on a ridge at *Q* = 0.1 − 0.15 Å^−1^, where 
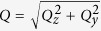
. For the AuNPs of bare surface, the ridge is on a broken ring of radius *Q* ≈ 0.13 Å^−1^, along which there are two intensity maxima at *Q*_*z*_ ≈ 0.02 and 0.12 Å^−1^. For the AuNPs coated with DNA, the ridge almost coincides with a ring of radius *Q* ≈ 0.12 Å^−1^.

**Figure 4:**
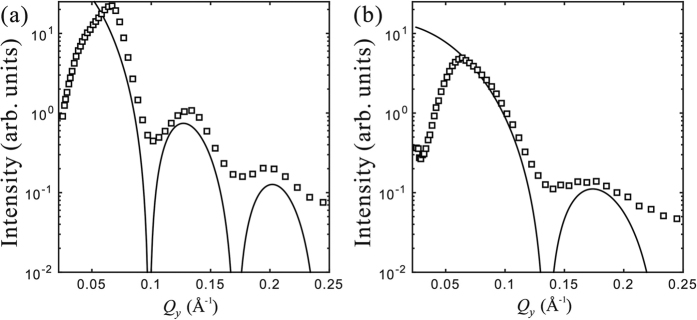
GISAXS intensity 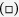 as function of *Q*_*y*_ at *Q*_*z*_ = 0.02 Å^−1^ for DPTAP monolayers on subphases containing (**a**) AuNPs of nominal radius 50 Å and (**b**) AuNP of nominal radius 25 Å. The solid lines are the 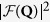 (vertically shifted for display purpose) for a solid sphere of radius 45 Å in (**a**) and 33 Å in (**b**) at *Q*_*z*_ = 0.02 Å^−1^ and *Q*_*x*_ ≈ 0.

Figure 4 shows the GISAXS intensity at *Q*_*z*_ ≈ 0.02 Å^−1^ that is enhanced significantly due the surface multiple scattering^17^,^24^. The intensity maxima coincide well with 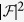 for a solid sphere of radius *R* that is close to the nominal radius of the AuNP. As the 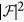 approach the maximum at *Q* = 0, the suppression of the intensity at low *Q*_*xy*_ indicates strong particle-particle interference arising from close packing^25^,^26^,^27^. The smearing of the sharp intensity minima present in the SAXS from monodisperse spheres is consistent with the polydispersity found in bulk SAXS shown in Fig. 1.

**Figure 5:**
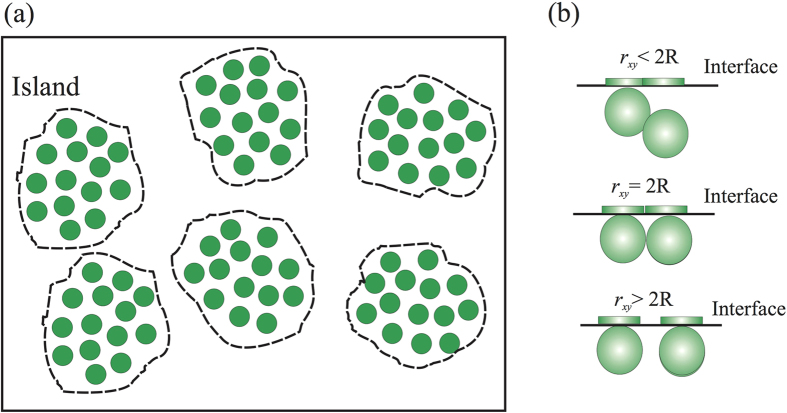
Surface covered by islands, each of which contains short range ordered, identical spheres. Island boundary is represented by the dashed line that encloses the spheres. The average surface density of spheres within an island is denoted as 〈*n*〉. (**b**) Schematic scenarios showing relative position of two particles and their corresponding projection onto the surface.

The quantitative GISAXS analysis is complex in general^24^. In this study, a simplistic, crude approximation, so called local monodisperse approximation (LMA), is adopted^25^,^28^. The LMA approximation states that the GISAXS intensity can be viewed as an incoherent sum of the intensities arising from coherently illuminated domains, each of which contains monodispersely distributed and spatially correlated particles, as illustrated in Fig. 5. Accordingly, the GISAXS intensity, *I*(*Q*_*xy*_, *Q*_*z*_), from one such domain consisting of identical spheres, can be expressed as^24^,^25^,^26^,^29^,^30^


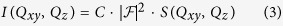


where *C* is an intensity scale factor. *S*(*Q*_*xy*_, *Q*_*z*_) represents the interference function of the spheres with the form factor 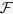 and is solely dependent on their relative positions^17^,^26^. *Q*_*xy*_ is the magnitude of the in-plane component of the scattering vector, and 
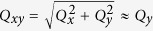
 as *Q*_*x*_ ≈ 0. Assuming all domains share the same *S*(*Q*_*xy*_, *Q*_*z*_) and replacing 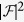 with 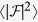 in Eq. (3), *I*(*Q*_*xy*_, *Q*_*z*_) thus can represent the GISAXS intensity averaged over all domains.

For distribution of particles in 3D space^17^,^26^, *S*(*Q*_*xy*_, *Q*_*z*_) is expressed as follows


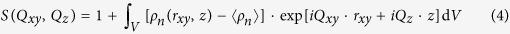


where *V* and d*V* represent the domain volume and the corresponding differential volume element, *ρ*_*n*_(*r*_*xy*_, *z*) the local number density of spheres at the (*r*_*xy*_, *z*) position, and 〈*ρ*_*n*_〉 the average number density of the spheres in the domain.

In the analysis, only the slice of intensity parallel to the surface is analyzed (i.e., at *Q*_*z*_ = 0.02 Å^−1^). As *Q*_*z*_ is very close to zero, Eq. (4) is rewritten as


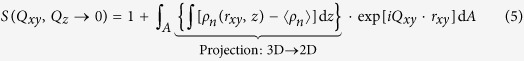


where *A* and d*A* represent the domain area and the corresponding differential area element. The integration enclosed in the curly brackets is the projection of the 3D density function onto the 2D plane (i.e., *x*-*y* plane), illustrated in Fig. 5(b). The range of projected distance between a pair of spheres in contact, *r*_*xy*_, is between zero and 2*R*, depending on the relative orientation.

Let *S*_2D_(*Q*_*xy*_) represent *S*(*Q*_*xy*_, *Q*_*z*_ → 0) and rewrite Eq. (5) as follows,


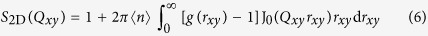


where the function J_0_(.) is the zero order Bessel function of the first kind and 〈*n*〉 is the average number of spheres per unit area. The product of *g*(*r*_*xy*_) and 〈*n*〉 gives the local number of spheres per unit area at a distance *r*_*xy*_ away from an arbitrarily chosen sphere center. The *g*(*r*_*xy*_) thus represents the 2D radial distribution function of spheres projected onto the plane.

In view of Eqs (3, 4, 5, 6), the GISAXS intensity at large *Q*_*xy*_ in Fig. 4 are clearly dominated by the form factor of the individual particles and *S*_2D_(*Q*_*xy*_) → 1, while at small *Q*_*xy*_, the intensity data is strongly suppressed by the *S*_2D_(*Q*_*xy*_). This feature in the 3D analog has been attributed to the short-range-order (SRO) among close-packed hard spheres that model liquid structures long ago by Kirkwood *et al*. via simulations^26^,^31^. Still, a general, explicit formula of structure factor for liquid-like structures is not readily available as the corresponding radial distribution function is determined by interaction potentials among particles that are in general difficult to ascertain^26^,^31^. Only a few structure factors are analytically available for certain inter-particle potential approximations (e.g., hard-sphere potential), by solving the Ornstein-Zernike (OZ) equation using the Percus-Yevick (PY) approximation^32^,^33^. These explicit structure factors have been applied to 3D colloidal suspension systems, but rarely applied to an interfacial system. The determination of the structure factor for particles in liquid-like state confined in 2D space heretofore, stimulated by the fundamental study on 2D phase transition, still relies on numerical computations^34^.

Another approach to deduce the structure factor is in the crystallographic manner by introducing disorders/imperfections into ideal crystal systems^35^,^36^. Lazzari and Renaud *et al*. investigated similar features in GISAXS patterns from solid surfaces^27^ and provided an empirical formula for *S*_2D_(*Q*_*xy*_)^29^. In a separate study, they also proposed a theoretical framework based on a classical 1D model (i.e., one-dimensional chain of correlated particles) to incorporate the particle size and spacing correlation into GISAXS analysis^25^.

In this study, we start with the radial distribution function and further introduce disorder effects. The details are presented in the Supporting Information (SI). The structure factor only includes a contribution from a few neighboring shells, which usually is the case for a liquid-like structure. The derived *S*_2D_(*Q*_*xy*_), denoted as *S*^SRO^(*Q*_*xy*_), is expressed as follows,


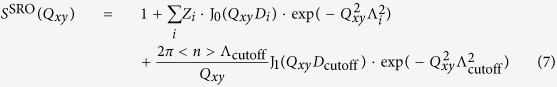


where *Z*_*i*_ and *D*_*i*_ are the coordination number and average 2D-projected distance of the *i*-th neighboring-shell (or annulus) around a central sphere. Λ_*i*_ is the 2D-projected, spatial spread of the *i*-th shell. J_1_(.) is the first order Bessel function of the first kind. *D*_cutoff_ is the 2D-projected distance where the crystal-like structure transits to the gas-like structure (i.e., ordered structure to an utterly disorder) occurs and Λ_cutoff_ being the associated uncertainty in the boundary of order-to-disorder transition. Let Λ_*i*_ = 0, Eq. (7) is a 2D analog of the Debye formula for 3D powder diffraction that is commonly used to calculate the scattering from small crystals containing a few building units^26^.

**Figure 6:**
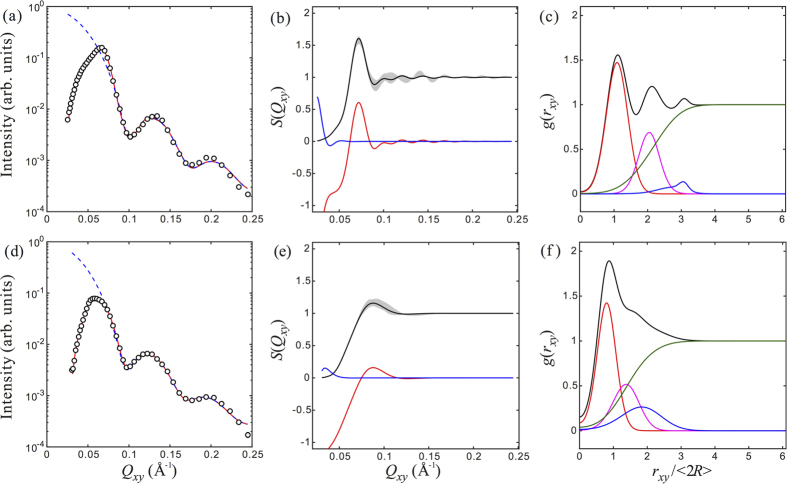
Data analysis for GISAXS intensity GISAXS line-cut intensity parallel to the surface (i.e., along *Q*_*y*_-axis) for AuNPs without DNA-coating (top row) and AuNPs with DNA-coating (bottom row). (**a**,**d**) GISAXS line-cut intensity data (circles) obtained at *Q*_*z*_ = 0.02 *Å*^−1^. The dashed lines are calculated, independent scattering intensity (i.e., 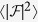) for a set of hypothetically uncorrelated spheres of given size distribution given in Table 1. The solid lines result from the multiplication of the 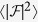 and the structure factor *S*_2D_(*Q*_*xy*_) shown in (**b**,**e**). (**b**,**e**) The ensemble averaged, positional structure factor *S*_2D_(*Q*_*xy*_). The shaded area indicates the variations of the local *S*_2D_(*Q*_*xy*_) profiles. The red lines are contributions from the countable (i.e., three) neighbor shells. The blue lines are contribution from the gas-like disorder term. (**c**,**f**) are radial distribution function directly converted from the ensemble averaged *S*(*Q*_*xy*_) in (**b**,**e**). The red, magenta and blue lines are contributions from the first, second and third neighboring shells. The green lines are contribution from the gas-like disorder term.

Figure 6 shows analysis of the data shown in Fig. 3 for AuNPs without DNA-coating (top row) and AuNPs with DNA-coating (bottom row), based on Eq. (7). The data shown in (a,d) are profile-fit with *S*_2D_(*Q*_*xy*_) (given in Eq. (7)) multiplied by 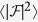 which has been obtained from the bulk SAXS data analysis. The corresponding profiles for *S*_2D_(*Q*_*xy*_) are shown in (b,e). The *S*_2D_(*Q*_*xy*_) profiles exhibit a main interference peak within *Q*_*xy*_ = 0.05 − 0.1 Å^−1^, for surface NP assemblies. The main interference peak for AuNP in the absence of surface DNA-coating appears more pronounced than in the presence of DNA-coating. Indeed, the main interference peak within the *Q*_*xy*_ window is mainly composed of the contributions from the neighboring shells, rather than the gas-like structure. In (c) and (f), the corresponding *g*(*r*_*xy*_) profiles are displayed, where the contribution from “crystal-like structure” and “gas-like structure” are shown. It appears that one can still identify the nearest neighbor and/or next nearest neighbor shells for bare AuNPs. In contrast, it is impossible to distinguish the lst and 2nd nearest neighbor shells for AuNP coated with DNA. If we assume *S*(*Q*_*xy*_, *Q*_*z*_) = *S*_2D_(*Q*_*xy*_) and include the multiple scattering effects, we can regenerate the full GISAXS map, as shown in Fig. 3(c,d) for both samples. The similarity between the measured GISAXS patterns in Fig. 3 and the calculations are pretty good indicating the *Q*_*z*_ dependence in *S*(*Q*_*xy*_, *Q*_*z*_) is nearly constant within the *Q*_*z*_ range measured. A complete *Q*_*z*_-independence corresponds to an absolute Dirac-delta *δ*(*z*) distribution of spheres. Our analysis also shows that the gas-like disorder term is not essential in fitting data within the current *Q*_*xy*_ range. This can be seen in Fig. 6(b,e) where the corresponding contribution from the gas-like disorder term is limited to extremely small *Q*_*xy*_, and almost zero at large *Q*_*xy*_. In Table 2 we list the structural parameters that are used to generate the model calculations shown in Fig. 6. These same parameters are used to simulate the 2D GISAXS patterns (Fig. 3(c,d)) that capture the experimental features adequately. We note that this approach to analyzing the GISAXS for general 2D systems with SRO is in fact the 2D analog to the Debye formula for 3D XRD of finite size crystals^26^.

## Conclusion

Using surface sensitive diffraction methods, i.e., X-ray reflectivity and grazing incidence X-ray small-angle scattering, we demonstrate that bare (unfunctionalized) gold nanoparticles (AuNP) are negatively charged and spontaneously adsorb to a cationic lipid template formed by a Langmuir monolayer (in this case formed by DPTAP (1,2-dihexadecanoyl-3-trimethylammonium-propane). The XRR yields the density profile across the charged-surface normal and shows that the AuNPs assemble as a single layer that is contiguous to the DPTAP monolayer. The analysis of the GISAXS in terms of a structure factor of loosely packed 2D particles indicates that the adsorbed AuNP monolayer exhibits short-range in-plane correlations. By contrast single-stranded-DNA-functionalized AuNPs, while attracted to the positively charged surface (more efficiently with the addition of NaCl to the solution) display less regular two-dimensional packing compared to bare AuNPs. Our results and analysis demonstrate that the behavior of AuNPs can be manipulated in a similar fashion to manipulating negatively charged ions. This opens a new avenue for assembly of nanoparticles by designing charged templates. Exploiting electrostatic forces is also relevant to functionalization of nanoparticles with charged entities such as polyelectrolytes to form shells with diverse functionalities. In addition, we provide a new approach to analyzing the GISAXS data for short-range-ordered particles in two-dimensions that directly yields the 2D pair-distribution function.

## Methods

### Reagents and Materials

1,2-dihexadecanoyl-3-trimethylammonium-propane (DPTAP) was purchased from Avanti Polar Lipids, Inc as powders and dissolved in 3:1 chloroform/methanol solution for Langmuir monolayer deposition. Gold nanoparticles with nominal size of 5 and 10 nm in diameter were purchased from Ted Pella, Inc and their actual size was determined by small-angel X-ray scattering (SAXS). Non-complementary single-stranded DNA capped gold nanoparticles (DNA-AuNPs) were synthesized according to the documented procedures in the literature with slight modifications^37^,^38^,^39^,^40^. The 5′-thiolated single-stranded DNA with 28 bases (sequence: 5′-SH-(CH_2_)_6_-TTT TTT GTG GAA AGT GGC AAT CGT GAA G-3′) was purchased from Integrated DNA Technologies as disulfides. The DNA with disulfide bonds was cleaved with dithiothreitol (Pierce Biotechnology, Thermo Scientific), purified with NAP-5 column (Sephadex G-25 DNA grade, GE Healthcare) and incubated with AuNPs solutions (nominal size 10 nm) under shaking at room temperature. The mixture of freshly cleaved DNA and AuNPs was then buffered with sodium phosphate (pH 7.0) followed by a slow salting process, in which the concentration of NaCl in the mixtures was gradually increased to 0.5 M over 2 days. The final mixture was aged at room temperature for another day and washed with Millipore water (18.2 MΩ · cm) by centrifugation (20000 *g* × 1 h) at least three times. DNA-AuNPs were dispersed in Millipore water and the concentration was determined with a UV-visible spectrophotometer. Unfunctionalized and DNA functionalized AuNPs at concentrations of 9 and 1 nM were used for the X-ray measurements, respectively.

### Experimental setup

**Figure 7:**
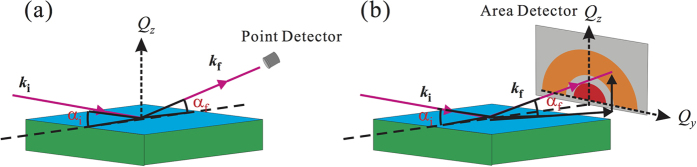
Schematic of setup for X-ray (**a**) reflectivity and (**b**) grazing incidence small-angle scattering.

Grazing incidence small-angle X-ray scattering (GISAXS) and reflectivity (XRR) measurements were carried out on the liquid surface spectrometer (LSS) at the at the 9-IDB beamline Advanced Photon Source (APS), Argonne National Laboratory. The highly monochromatic X-ray beam of energy *E* = 13.474 keV (corresponding wavelength *λ* = 0.9201 Å) yields a critical incident-angle for total reflection, *α*_c_ = 0.0915°. A schematic illustration of the X-ray experimental setup is shown in Fig. 7. For an XRR measurement, a Bicron point detector is used to collect reflected beam at an exit angle *α*_f_ = *α*_i_ in the scattering plane (defined by the surface normal (*z*-axis) and the incident beam). The reflectivity is measured as a function of the *z*-component of the scattering vector **Q**, i.e. *Q*_*z*_ = (4*π*/*λ*) sin *α*_i_. For a GISAXS measurement, a digital, two-dimensional Pilatus 100 K detector (487 × 195 pixels, 172 × 172 *μ*m per pixel) has been placed 2041 mm away (downstream) from the aqueous surface (sample) center. The scattering vector, **Q**, for GISAXS data, is calibrated with a diffraction pattern obtained from a standard silver-behenate powder. The three components of the scattering vector, i.e., (*Q*_*x*_, *Q*_*y*_, *Q*_*z*_), are normal to one another and are defined in such way that the *Q*_*z*_ component is along the surface normal while *Q*_*x*_ and *Q*_*y*_ components are within the surface. For GISAXS, *Q*_*y*_ is conventionally defined as parallel to the surface of detector for *Q*_*x*_ ≈ 0. Therefore, the magnitude of the in-plane scattering vector, *Q*_*xy*_, defined as 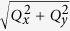, is approximately equivalent to *Q*_*y*_. Analysis methodology is provided in detail in the SI and elsewhere^41^,^42^. The DPTAP monolayer is spread on aqueous nanoparticle suspension that is contained in an enclosed thermostated Teflon Langmuir trough (dimension 60 mm by 60 mm). The monolayer compression is performed with a motorized Teflon bar and surface pressure is measured with a Wilhelmy plate. The trough canister is sealed and purged with water-saturated helium gas in which the oxygen level is monitored with a sensor (S101, Qubit System Inc.) during the X-ray measurements (waiting time before measurements 0.5–1 hour). The temperature in the trough is kept constant at 20 °C.

Small-angle X-ray scattering (SAXS) in transmission mode has been performed at beamline 12ID-B, APS, on dilute AuNP bulk solutions to determine the actual size and distribution of the particles in the suspension. For this experiments, the AuNPs solutions were loaded in a flow cell that is vertically mounted normal to the incident X-ray beam (X-ray energy *E* = 14.0 keV). A 2D detector Pilatus2m was used and the scattering vector magnitude *Q* was calibrated with silver behenate powder low-angle diffraction. Various concentrations of AuNPs were examined to ensure independent scattering from particles. The 2D SAXS images were then converted to 1D data through radial average and further corrected for background subtraction and intensity normalization^19^.

## Additional Information

**How to cite this article**: Wang, W. *et al*. Assembling Bare Au Nanoparticles at Positively Charged Templates. *Sci. Rep*. **6**, 26462; doi: 10.1038/srep26462 (2016).

## Supplementary Material

Supplementary Information

## Figures and Tables

**Table 1:** Particle size distribution determined with model refinement of SAXS data in terms of Eq. (2).

Nominal diameter(Å)^a^	2〈*R*〉 (Å)	2Δ*R* (Å)
100	89	8
50	65	10

^a^Nominal diameters of the AuNPs are provided by the manufacturer Ted Pella, Inc.

**Table 2:** Structural parameters determined by GISAXS^a,^^b,^^c^.

Samples	Bare AuNPs	DNA-AuNPs
*Z*_1_	9.0 ± 1.1	6.0 ± 1.4
*D*_1_ (Å)	99 ± 2	76 ± 2
Λ_1_/*D*_1_	0.2	0.2
*Z*_2_	8 ± 3	4.9 ± 1.2
*D*_2_/*D*_1_	1.8 ± 0.1	1.7 ± 0.1
Λ_2_/*D*_1_	0.2	0.3
*D*_cutoff_/*D*_1_	2.0 ± 0.2	1.8 ± 0.3
Λ_cutoff_/*D*_1_	0.4 ± 0.1	0.5 ± 0.1

^a^Inclusion of the parameter *Z*_3_, *D*_3_ and Λ_3_ can improve the profile fitting, but is not essential.

^b^The data fitting is not sensitive to the parameter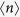. Thus the 〈*n*〉 is allowed to vary in the 1.5 ± 0.5 × 10^−4^ Å^−2^ range.

^c^The contribution of gas-like disorder is mainly restricted to low *Q*_*xy*_ regime. The larger the *D*_cutoff_, the lower the *Q*_*xy*_ regime it dictates. So only the lower limit of the *D*_cutoff_ is considered in this study.
