# Supplementary material for: Assembling Bare Au Nanoparticles at Positively Charged Templates
Source: Sci Rep. 2016 May 26;6:26462. doi: 10.1038/srep26462 (PMC4881021; doi:10.1038/srep26462)
Supplement: Supplementary Information [file srep26462-s1.pdf]

Assembling Bare Au Nanoparticles at Positively Charged Templates

Wenjie Wang, Honghu Zhang, Ivan Kuzmenko, Surya Mallapragada and David Vaknin

Supporting information

Here, we set out to construct a 2D radial distribution function, $g(r_{xy})$, following Eq. (5) and Eq. (6), for a collection of N rigid spheres of radius R confined in a 2D domain of area A. We begin with the expression for most ordered 2D lattice strictly confined in a plane and then modify it to suit a less stringent, amorphous case in planar morphology.

The positions of spheres are represented by the coordinates of their centers of mass. The cylindrical coordinate system, where z-axis is oriented along the normal of the plane, is adopted. One of the spheres is arbitrarily chosen as the origin and the relative positions of other spheres are denoted as ($\mathbf{r}_{xy}$, z), where the vector $\mathbf{r}_{xy}$ is the position vector in x-y plane. Taking into account the orientational average of the domain within the x-y plane, the local number density of the spheres, $\rho_n(\mathbf{r}_{xy}, z)$, depends only on the distance r_{xy}, i.e., $\rho_n(\mathbf{r}_{xy}, z) = \rho_n(r_{xy}, z)$. To model an assembly of AuNPs adorbed at the air-water interface, the z-coordinates of all spheres only vary over a very finite range around an average position $\langle z\rangle$, and $\langle z\rangle = 0$. The surface density of spheres, denoted as $n(r_{xy})$, are thus $n(r_{xy}) = \int \rho_n(r_{xy}, z)\mathrm{d}z$. Let $\langle n\rangle$ denote the average surface density, which equals to N/A, then $n(r_{xy}) = \langle n\rangle g(r_{xy})$.

To begin with, we consider the case of regularly packed spheres with their centers of mass confined in the x-y plane (i.e., $z = 0$). The density can be expressed as $\rho_n(r_{xy}, z) = n(r_{xy}) \cdot \delta(z)$, where $\delta(z)$ is the Dirac-delta function and surface density $n(r_{xy})$ bears the discrete characteristics of a 2D lattice (e.g., hexagonal and square lattice) representing long-range-order (LRO). The corresponding $g(r_{xy})$, denoted as $g^{\mathrm{LRO}}(r_{xy})$, features a series of well-defined, sharp peaks, each of which corresponds to a shell (or ring; we use shell and ring interchangeably) of spheres relative to the sphere at the origin. For instance, for a hexagonal lattice, the first peak is located at $D_1 = 2R$, the second peak at $D_2 = 2\sqrt{3}R$, ..., where D_i represents the i-th shell location around the sphere at the origin.[31] Similar to the Debye formula for powder patterns,[26] $g^{\mathrm{LRO}}(r_{xy})$ is a sum of contributions from each individual shell,[41] as follows

$$g^{\mathrm{LRO}}(r_{xy}) = \sum_{i=1}^{\infty} g_i(r_{xy}) \tag{S1}$$

where

$$g_i(r_{xy}) = \frac{Z_i \delta(r_{xy} - D_i)}{2\pi r \langle n\rangle} \tag{S2}$$

Z_i ($i = 1, 2, \ldots$) are the coordination number of the i-th shell around the sphere at the origin. $Z_i = 2\pi\langle n\rangle \int_0^{\infty} g_i(r_{xy}) r_{xy} \mathrm{d}r_{xy}$. For an ideally infinite sized crystalline body, the right hand side (RHS) of Eq. (S1) consists of infinite terms.

For a 2D liquid-like (short range order) structure, the $g(r_{xy})$, denoted as $g^{\mathrm{SRO}}(r_{xy})$, is somewhere between the LRO manifested as $g^{\mathrm{LRO}}(r_{xy})$ and utterly disorder manifested as $g(r_{xy}) = 1$ for a gas-like structure.[17,18,31]

$$g^{\mathrm{SRO}}(r_{xy}) = \underbrace{\sum_{i=1} g_i(r_{xy})}_{\text{Crystal-like structure}} + \underbrace{g_{\mathrm{cutoff}}(r_{xy})}_{\text{Gas-like structure}} \tag{S3}$$

where

$$g_{\mathrm{cutoff}}(r_{xy}) = \begin{cases} 1, \text{if } r_{xy} > D_{\mathrm{cutoff}} \\ 0, \text{if } r_{xy} < D_{\mathrm{cutoff}} \end{cases} \tag{S4}$$

Equation S3 states that only finite terms are included accounting for finite correlation length and an additional cutoff term (with the subscript ”cutoff”) is included accounting for the complete disorder. Beyond a cutoff distance (comparable to the correlation length), D_{cutoff}, the local density is simply replaced with $\langle n\rangle$, i.e., $g(r_{xy}) = 1$. In view of the fact that the correlation length spans only over a few immediate neighboring shells the cutoff length D_{cutoff} becomes comparable to a few sphere diameters as is typical for a liquid structure.[26,31]

Next step is to consider the ”smearing” of the distribution of spheres in the neighboring shells to account for the scenario shown in Fig.5, i.e., the spheres are allowed to deviate from the 2D close packing in x-y plane or deviate from $z = 0$ in the z direction. A Gaussian probabilistic function, is used to spread out the Dirac-delta functions to provide definitive distribution of spheres in each shell through the convolution operation as follows.

$$g^{\mathrm{SRO}}(r_{xy}) = \sum_i g_i \otimes P_i(r_{xy}) + g_{\mathrm{cutoff}} \otimes P_{\mathrm{cutoff}}(r_{xy}) \tag{S5}$$

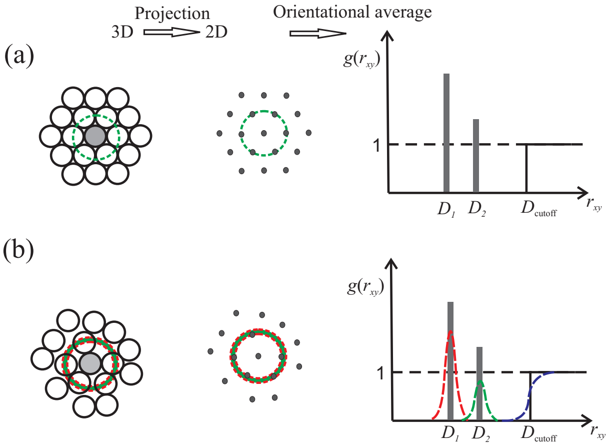

Figure S1. The radial distribution function for (a) a 2D crystalline array of spheres and (b) a simple liquid-like, disordered array of spheres. The dashed line in (a) indicates the definite positions of first coordination shell of an arbitrarily chosen sphere. In liquid-like structure, the first coordination shell is less definite, and is located in an annulus representing the probable location of the first coordination shell.

.

where the symbol '⊗' denotes the convolution operator in 2D for functions of cylindrically symmetry. $P_i(r_{xy})$ is the Gaussian probabilistic function defined as

$$P_i(r_{xy}) = \frac{1}{2\pi\Lambda_i^2}\exp\left(-\frac{r_{xy}^2}{2\Lambda_i^2}\right)$$
$$P_{\mathrm{cutoff}}(r_{xy}) = \frac{1}{2\pi\Lambda_{\mathrm{cutoff}}^2}\exp\left(-\frac{r_{xy}^2}{2\Lambda_{\mathrm{cutoff}}^2}\right)$$

where Λ_i is the measure of the spatial spread of the the location of the i-th shell and $\Lambda_{\mathrm{cutoff}}$ the range over which the SRO transits into utterly disorder, i.e., $g(r_{xy}) = 1$.[26,31]

The structure factor for the SRO, based on the $g^{\mathrm{SRO}}(r_{xy})$, upon the Hankel transform of Eq. (6),[42] is expressed as

$$S^{\mathrm{SRO}}(Q_{xy}) = 1 + \sum_i Z_i \cdot \mathrm{J}_0(Q_{xy}D_i) \cdot \exp(-Q_{xy}^2\Lambda_i^2) + \frac{2\pi < n > \Lambda_{\mathrm{cutoff}}}{Q_{xy}}\mathrm{J}_1(Q_{xy}D_{\mathrm{cutoff}}) \cdot \exp(-Q_{xy}^2\Lambda_{\mathrm{cutoff}}^2) \quad \text{(S6)}$$

The gaussian terms account for the positional fluctuations, the analog of the Debye-Waller factor in X-ray diffraction.[17,18]
